# Knowledge, attitude and associated factors towards end of life care among nurses’ working in Amhara Referral Hospitals, Northwest Ethiopia: a cross-sectional study

**DOI:** 10.1186/s13104-019-4567-7

**Published:** 2019-08-19

**Authors:** Addisu Taye Abate, Fisseha Zewdu Amdie, Netsanet Habte Bayu, Dawit Gebeyehu, Tesfamichael G/Mariam

**Affiliations:** 1 Department of Medical Nursing, School of Nursing, College of Medicine and Health Sciences, University of Gondar, Gondar, Ethiopia; 2 Department of Surgical Nursing, School of Nursing, College of Medicine and Health Sciences, University of Gondar, Gondar, Ethiopia; 3 Department of Midwifery, College of Medicine and Health Sciences, University of Gondar, Gondar, Ethiopia

**Keywords:** Knowledge, Attitude, Nurses, End of life care and Ethiopia

## Abstract

**Objective:**

The aim of this study was to assess nurses’ Knowledge, Attitude and Associated Factors towards end of life care in Amhara Referral Hospitals, Northwest Ethiopia, 2017.

**Results:**

A total of 331 participants were included with a response rate of 93.2%. From these, 129 (39.0%) of them had good knowledge and 234 (70.7%) had favorable attitude towards end of life care. Being Bachelor of Science holder and above in nursing (AOR = 4.261, 95% CI 1.524–11.912), working in Emergency department (AOR = 4.911, 95% CI 1.796–13.426), having daily experience of caring for chronically ill patients (AOR = 2.764, 95% CI 1.366–5.591) and taking training on end of life care (AOR = 10.269, 95% CI 4.730–22.296) were significantly associated with good knowledge of nurses towards end of life care. On the other hand, having 6–10 years of working experience in nursing (AOR = 2.199, 95% CI 1.147–4.215), being trained in end of life care (AOR = 3.027, 95% CI 1.285–7.13), Bachelor of Science holder and above in nursing (AOR = 4.414, 95% CI 2.230–8.738) were found to be statistically significant with more positive attitude of nurses towards end of life care.

**Electronic supplementary material:**

The online version of this article (10.1186/s13104-019-4567-7) contains supplementary material, which is available to authorized users.

## Introduction

End-of-life care (EoLC) refers to all aspects of the care relating to dying, death and bereavement which are provided towards the end of life [1]. EoLC prevent or relieve suffering through management of pain or other symptoms and provision of psychological, social, spiritual and physical support. Nowadays, the place where people die is moving to hospital settings due to a value in saving and prolonging life using advanced medical technologies [2]. Globally, there is 57.4 million deaths per year [3]; and many of these deaths were accompanied by suffering that needs quality and compassionate EoLC.

However, EoLC has been identified as an area where quality of care has previously been “very variable” [4], there is limited development of care for the dying patients in Africa and only a small proportion of people in the community receive end-of-life care in specialized settings like cancer treatment and any other rehabilitation centers [5, 6]. In parallel with population ageing the social environment is also rapidly changing; the size and role of families/extended families is decreasing; and trends in support and caring for the dying person are rapidly changing [7]. These issues will further increase the demand for care at the end-of-life in health care settings.

At present, too many Ethiopians are experiencing uncertainty, pain and suffering in the final months and days of their lives [8, 9]. Despite this, there are no government programs offering hospice care [10]. Managing EoLC has recently been identified as a priority in nursing, although there are some barriers to deliver it. From these, absence of appropriate education and training for staff is the single most important factor [11–14]. However, WHO has developed guidance on the integration of palliative care into existing health system at all levels [15].

Despite of the fact that Nurses play a key role in caring for dying patients [12], a modified systematic review in Cameroon revealed that nurses describe themselves as lacking confidence in providing care for the dying and rate the extent of their education on EoLC as ‘limited’ [13]. A systematic review article describes nurse’s personal characteristics (age, years of work experience in nursing, EoLC training, educational level and direct experience in taking care of the dying patient or family member) as personal factors which are associated with better knowledge of end of life care, and more positive attitudes toward EoLC [16].

As far as our knowledge is concerned, there is no sufficient research specifically on nurses’ EoLC knowledge and Attitude in the northwest part of Ethiopia. Thus, the documentation of existing knowledge and attitudes of nurses towards EoLC would help in planning and integrating the necessary interventions into continuing education sessions and routine assessments according to areas of weakness. Therefore, the purpose of this study is to assess nurses’ knowledge and attitude toward EoLC with its associated factors in Amhara region, Northwest of Ethiopia referral Hospitals.

## Main text

### Study design and setting

Institutional based cross-sectional study was conducted from April 01–15, 2017 in Amhara region Referral Hospitals, Northwest Ethiopia.

### Sample size determination and procedure

The source population was all nurses who were working in Amhara Referral Hospitals, Northwest Ethiopia. All permanently employed nurses and those who were working during the study period in the hospital were included; whereas, those nurses who were on annual leave, maternity leave and seriously ill during the time of data collection were excluded from the study. By means of single population proportion formula; 95% level of confidence with 5% margin of error and 10% non-response rate were considered to estimate the required sample size of 355 nurses. Systematic random sampling technique with a sampling interval “K” value of Three, which was calculated as K = N/nf, where N = total number of nurses = 949 and nf = final sample size = 355 was employed. Thus, by using list of nurses as a sampling frame, participants were selected in every 3 number intervals till to attain the required sample size and the first participant was selected by computer generated lottery method. Participants’ involvement in the study was on voluntary basis and they were not offered any incentive. Finally, nurses were invited to participate in the study by using a face-to-face method of self-administered questionnaire.

### Data collection method and instrument

Data collection was carried out by three BSc nurses; who were properly trained for 3 days regarding the questionnaire. Initially, the questionnaire was translated from English to Amharic, which is local language of the region; then it was re-translated to English language to ensure its consistency. The tool, which is structured self-administered questionnaire was pretested on 36 nurses; and they were not included in the final analysis. The knowledge questionnaire adapted from the End of Life Knowledge Assessment for Nurses; it uses 24 items with multiple choice format and those who score 50% and above it were considered as having good knowledge [17]. The attitude questionnaire adapted from From-melt Attitude toward Care of the Dying (FATCOD) which consists 30 items. It has a 5 point Likert scale; i.e. (1) Strongly Disagree, (2) Disagree, (3) Uncertain, (4) Agree and (5) Strongly Agree. Positive items were scored from 1 for Strongly Disagree to 5 for Strongly Agree. For negative items the scoring was reversed. Finally, the number of items on which participants either agreed or strongly agreed (or disagreed/strongly disagreed for negatively worded items) were calculated for each participant and those study participants who score ≥ 50% of the total score were considered to have favorable attitude. Both the knowledge and attitude questionnaires used several times to assess nurses’ knowledge and attitudes toward care of the dying [17–19].

### Data processing and analysis

Data were cleaned; entered and analyzed using Epi info version 7 and SPSS Version 23 respectively. Study participants were described by using percentages, mean and median with standard deviation. Binary logistic regression analysis was computed for all variables to identify factors associated with good knowledge and more positive attitude towards EoLC. All independent variables with p < 0.2 in bivariate analysis were again entered to multivariable logistic regressions to control the effect of confounding and significant factors were identified based on Adjusted Odds Ratio (AOR) included in 95% confidence level at *p* value less than 0.05. Missing data were treated by deleting the observations (missing values were not included during model building).

### Results

#### Socio-demographic characteristics of the study participants

Out of the total sample size (n = 355), 331 participants have responded completely resulting in a 93.2% response rate. Among these, almost half of the participants 186 (56.2%) were female and the mean age of respondents was 29.2 years ± 4.499 SD (ranged from 23 to 46) (Table 1).

**Table 1:** Socio-demographic characteristics of nurses in Amhara region Referral Hospitals, Northwest Ethiopia, 2017 (N = 331)

Variables	Responses	Frequency (N = 331)	Percentage
Institution/hospital	Hospital 1	185	55.9
	Hospital 2	96	29.0
	Hospital 3	50	15.1
Age	23–30 years	225	68.0
	31–40 years	90	27.2
	> 40 years	16	4.8
Sex	Male	145	43.8
	Female	186	56.2
Religion	Orthodox	260	78.5
	Muslim	40	12.1
	Protestant	31	9.4
Monthly income (ETB)	≤ 5000	102	30.8
	> 5000	229	69.2
Marital status	Never married	205	61.9
	Married	123	37.2
	Divorced	3	0.9
Level of education	Nursing diploma	45	13.6
	Bachelors in nursing	272	82.2
	Masters in nursing	14	4.2
Current working area	Medical	50	15.1
	Surgical	36	10.9
	Outpatient department	60	18.1
	Pediatric	61	18.4
	Post anesthesia/recovery	10	3.0
	Intensive care unit	4	1.2
	Ophthalmology	11	3.3
	Emergency room	29	8.8
	Obstetrics and gynecology	36	10.9
	Operation Theater	24	7.3
	Central Supply	10	3.0
Years of working experiences in nursing	≤ 5 years	230	69.5
	6–10 years	77	23.3
	11–15 years	11	3.3
	> 15 years	13	3.9
Experience in caring chronically ill patient	Daily	153	46.2
	Once per week	78	23.6
	Once per month	48	14.5
	Few times per year	4	1.2
	Never	48	14.5
Experience of care for a dying family member	Yes	107	32.3
	No	224	67.7
In-service training about End of life	Yes	62	18.7
	No	269	81.3

*ETB* Ethiopian Birr

#### Nurses’ knowledge and attitude towards EoLC

Based on nurses’ End of Life Knowledge Assessment tool, the minimum and maximum scores were 4 and 22 out of 24 respectively, with the mean score of 11.49 ± 4.108 SD. About 153 (46.2%) of the respondents knew the goals of EoLC and more than half (56.5) of participants said that the most accurate judge of the intensity of the patient’s pain is the patient himself. Regarding to nurses’ attitude, it appears that approximately 40% of the respondents believed to change the subject to something cheerful when a patient asks a nurse, “Am I dying?”, and nearly half (48.9%) of them do not believe that providing care to the dying person is a worthwhile experience. In the other hand, over 50% of nurses did not think that it was possible for non-family caregivers to assist a patient to prepare for death; and majority of them (71.9%) were comfortable to talk about death with dying patients (see Additional file 1: Table S1). In general, Out of the total study participants only 129 (39.0%) of them have good knowledge; whereas, most of the respondents’ 234 (70.7%) have favorable attitude towards EoLC with the mean score of 92.98 ± 17.208 SD.

#### Factors associated with nurses’ knowledge and attitude towards EoLC

In the present study, Nurses with BSc degree and above level of education were more knowledgeable (AOR = 4.261, CI 1.524–11.912, P = 0.006) and had more favorable attitude (AOR = 4.414, CI 2.230–8.738; p ≤ 0.001) than diploma holders. Similarly, Nurses who took training on EoLC had more knowledge (AOR = 10.269, CI 4.730–22.296; P ≤ 0.001) as well as they have more positive attitude (AOR = 3.027, CI 1.285–7.130; p = 0.011) compared to nurses who did not take in-service training. Moreover, working in Emergency department, having experience in caring chronically ill patient and their family member affected EoLC knowledge positively. On the other hand nurses who had 6–10 years of working experience in nursing (AOR = 2.199, CI 1.147–4.215, p = 0.018) had more favorable attitude than those nurses—with ≤ 5 years of working experience (Tables 2, 3).

**Table 2:** Factors associated with knowledge of Nurses towards EoLC in Amhara region Referral Hospitals, Northwest Ethiopia, 2017 (N = 331)

Factors	Knowledge		COR 95 (CI)	AOR 95 (CI)	p-value
	Good (no)	Poor (no)			
Institution					
Hospital 1	66 (35.7%)	119 (64.3%)	1.381 (0.687–2.775)		
Hospital 2	44 (45.8%)	52 (54.2%)	0.905 (0.475–1.725)		
Hospital 3	19 (38.0%)	31 (62.0%)	1		
Sex					
Male	52 (35.9%)	93 (64.1%)	1		
Female	77 (41.4%)	109 (58.6%)	1.263 (0.808–1.977)		
Age					
23–30	94 (41.8%)	131 (58.2%)	1		
31–40	31 (34.1%)	60 (65.9%)	1.973 (0.610–6.387)		
> 40	4 (26.7%)	11 (73.3.0%)	1.421 (0.418–4.831)		
Religion					
Orthodox	104 (40.0%)	156 (60.0%)	1		
Muslim	12 (30.0%)	28 (70.0%)	0.643 (0.313–1.321)		
Protestant	13 (41.9%)	18 (58.1%)	1.083 (.509–2.306)		
Educational					
Diploma	6 (13.3%)	39 (86.7%)	1		
BSc. and above	123 (43.0%)	163 (57.0%)	4.905 (2.013–11.95)	4.261 (1.524–11.91)	0.006*
Marital status					
Single	89 (42.0%)	123 (58.0%)	1		
Married	40 (33.6%)	79 (66.4%)	1.429 (0.895–2.282)		
Clinical area					
OPD	18 (22.5%)	62 (77.5%)	1		
Emergency	27 (40.3%)	40 (59.7%)	2.325 (1.135–4.761)	4.91 (1.796–13.426)	0.002*
Inpatient	84 (45.7%)	100 (54.3%)	2.893 (1.589–5.270)	5.85 (2.221–15.443)	0.000*
Working experience					
≤ 5	95 (41.1%)	136 (58.9%)	1		
6–10	24 (31.24%)	53 (68.8%)	0.648 (0.374–1.122)		
> 10	10 (43.5%)	13 (56.5%)	1.101 (0.464–2.616)		
Experience in caring chronically ill					
Daily	76 (49.7%)	77 (50.3%)	2.809 (1.624–4.858)	2.764 (1.366–5.591)	0.005*
Once per week	27 (34.6%)	51 (65.4%)	1.507 (0.790–2.875)	3.388 (1.364–8.415)	0.009*
Once/month and less	26 (26.0%)	74 (74.0%)	1		
Experience of caring for a dying family member					
No	64 (28.6%)	160 (71.4%)	1		
Yes	65 (60.7%)	42 (39.3%)	3.869 (2.384–6.280)	4.262 (2.390–7.603)	0.000*
EoLC training					
No	80 (29.7%)	189 (70.3%)	1		
Yes	49 (79.0%)	13 (21.0%)	8.905 (4.57–17.315)	10.26 (4.730–22.29)	0.000*
Monthly salary					
≤ 5000	28 (27.5%)	74 (72.5%)	1		
> 5000	101 (44.1%)	128 (55.9%)	2.085 (1.256–3.463)		

NB: variables having a (P ≤ 0.2) in bivariable (unadjusted) analysis included in the multivariable (adjusted) analysis

*AOR*, adjusted odds ratio, *COR* Crude Odds Ratio, *BSc* Bachelor in Science, *OPD* out patient department

*Statistically significant at p-value ≤ 0.05

**Table 3:** The association between socio-demographic characteristics and attitude of Nurses towards EoLC in Amhara region Referral Hospitals, Northwest Ethiopia, 2017 (N = 331)

Factors	Attitude		COR 95 (CI)	AOR 95 (CI)	p-value
	Fav. (no)	Unfav. (no)			
Institution					
Hospital 1	131 (70.8%)	54 (29.2%)	1.040 (0.525–2.058)		
Hospital 2	68 (70.8%)	28 (29.2%)	1.041 (0.493–2.199)		
Hospital 3	35 (70.0%)	15 (30.0%)	1		
Sex					
Male	107 (73.8%)	38 (26.2%)	1		
Female	127 (68.3%)	59 (31.7%)	0.764 (0.472–1.238)		
Age					
23–30	160 (71.1%)	65 (28.9%)	1		
31–40	63 (70.0%)	27 (30.0%)	0.895 (0.275–2.913)		
> 40	11 (68.8%)	5 (31.2%)	0.818 (0.240–2.793)		
Religion					
Orthodox	191 (73.5%)	69 (26.5%)	1		
Muslim	26 (65.0%)	14 (35.0%)	0.671 (0.331–1.359)		
Protestant	17 (54.8%)	14 (45.2%)	0.439 (0.205–0.937)		
Educational status					
Diploma	18 (40.0%)	27 (60.0%)	1		
BSc and above	216 (75.5%)	70 (24.5%)	4.629 (2.406–8.906)	4.41 (2.23–8.73)	0.000*
Marital status					
Single	150 (70.8%)	62 (29.2%)	1		
Married	84 (70.6%)	35 (29.4%)	1.008 (0.616–1.650)		
Clinical area					
OPD	53 (66.2%)	27 (33.8%)	1		
Emergency	46 (68.7%)	21 (31.3%)	1.116 (0.558–2.233)		
Inpatient	135 (73.4%)	49 (26.6%)	1.404 (0.796–2.475)		
Year of experience					
≤ 5	164 (71.6%)	65 (28.4%)	1		
6–10	53 (67.1%)	26 (32.9%)	1.944 (1.051–3.593)	2.19 (1.14–4.21)	0.018*
> 10	17 (73.9%)	6 (26.1%)	3.399 (0.98–11.789)	2.66 (0.72–9.83)	0.140*
Experience in caring chronically ill					
Daily	111 (72.5%)	42 (27.5%)	1.302 (0.753–2.251)		
Once per week	56 (71.8%)	22 (28.2%)	1.254 (0.657–2.391)		
Once/month and less	67 (67.0%)	33 (33.0%)	1		
Experience of caring for a dying family member					
No	150 (67.0%)	74 (33.0%)	1		
Yes	84 (78.5%)	23 (21.5%)	1.802 (1.051–3.088)	1.72 (0.96–3.07)	0.066
EoLC training					
No	179 (66.5%)	90 (33.5%)	1		
Yes	55 (88.7%)	7 (11.3%)	3.951 (1.729–9.026)	3.02 (1.28–7.13)	0.011*
Monthly salary					
≤ 5000	57 (55.9%)	45 (44.1%)	1		
> 5000	177 (77.3%)	52 (22.7%)	2.687 (1.633–4.423)		

*Fav.* favorable, *Unfav.* unfavorable, *NB* variables having a (0 ≤ 0.2) in bivariable (unadjusted) analysis included in the multivariable (adjusted) analysis

*AOR*, adjusted odds ratio, *COR* Crude Odds Ratio, *BSc* Bachelor in Science, *OPD* out patient department

*Statistically significant at p-value ≤ 0.05

### Discussion

The result of this study showed that only (39%) of nurses had good knowledge towards EoLC. This finding is consistent with the study done in Saudi Arabia and Addis Ababa in which (38% and 35.4%) of the respondents had good knowledge [20, 21] respectively. However, the finding of the present study lower than the study conducted in Korea and Nigeria (45.6% and 94.6%) of nurses had good knowledge respectively [18, 22]. The possible reason for this might be that only a few nurses (18.7%) have been trained on EoLC in the present study. In this regard, in service training for nurses on EoLC was the most frequently described professional need [23].

Regarding attitude, nearly half (49.6%) of the respondents in this study believe that providing care to the dying person is a worthwhile experience. The finding is consistent with studies done in Egypt (48.7%) [24]. Furthermore, (28.1%) of the nurses were uncomfortable to talk about death with dying patients and it was lower than the result from Egypt (43.7%) and higher than from South African (24%) [24, 25]. This difference might be because of cultural differences related to delivering bad news or talking about death in front of the patient. However, a study done in south-east Ira (94% to 99%) of the participant believes that it is important to tell terminal ill patients and their family about the diagnosis and prognosis of the patient [26].

Overall, majority (70.7%) of nurses had favorable attitude towards EoLC, and which is higher than a study conducted in Korea; in which only (45.2%) of nurses had favorable attitude towards EoLC [22]. This result may be related to the Nurses’ values and intrinsic religious beliefs. Conversely, this finding is lower than a study conducted in Iran, which was (81%) of the study respondents have positive attitude regarding EoLC [27].

The findings of this study showed being Bachelor of Science holder and above in nursing, having 6–10 years of working experience in nursing and taking training on EoLC were significantly associated with both good knowledge and more positive attitude of nurses towards EoLC. This finding supported by a study, which showed that factors related to a nurse’s caring behavior of a dying patient, including educational level, experience and training; and there was a significant increase in nurses’ knowledge and attitude compared to the time before training [24, 25, 27]. In addition, nurses who have more professional experience in caring chronically ill patient were described as more confident in managing dying patients’ symptoms [27, 28]. This suggests that those nurses with less educational status and experience needs additional training in this area.

### Conclusion

Nurses’ knowledge on EoLC found to be poor; but majority of them have favorable attitude. In conclusion, findings of this study revealed that nurses should be familiarized with the concepts of EoLC through trainings, workshops and formal or informal education in both academic and hospital settings. In addition, nursing schools in higher education institutions should also revise the curriculum to incorporate courses related to EoLC so as to strengthen their students’ level of understanding related to delivering care for the dying patients.

## Limitations of the study

Shortage of similar studies carried out in Ethiopia makes the comparison and discussion difficult. Similarly, instead of using the knowledge mean score, a range was considered to compare levels of knowledge with previous studies. Lack of qualitative assessment in the study restricted to rule out all the possible associated factors related to attitude of nurses on EoLC. Furthermore, since participants were selected systematically from the list of eligible nurses, it could be open to bias in case nurses with specific characteristics being selected.

## Additional file

**Additional file 1: Table S1.** Nurses’ attitude according to their degree of agreement toward items of FATCOD in Amhara region Referral Hospitals, Northwest Ethiopia, 2017.

## Data Availability

The datasets used and analyzed during the current study are available from the corresponding author on reasonable request.

## Abbreviations

CI: confidence interval
CMHS: College of Medicine and Health Science
EoLC: End of Life Care
Epi info: Statistical package for epidemiological information analysis
ETB: Ethiopian Birr
FATCOD: From melt Attitude toward Care of the Dying scale
FMDPs: family members of dying patients
NCDs: non-communicable diseases
PC: Palliative Care
PI: principal investigator
SD: standard deviation
SPSS: Statistical Package for Social Science
UoG: University of Gondar
USA: United States of America
WHO: World Health organization
