# Supplementary material for: Knowledge, attitude and associated factors towards end of life care among nurses’ working in Amhara Referral Hospitals, Northwest Ethiopia: a cross-sectional study
Source: BMC Res Notes. 2019 Aug 19;12:521. doi: 10.1186/s13104-019-4567-7 (PMC6700991; doi:10.1186/s13104-019-4567-7)
Supplement: Supplementary file 1 — Additional file 1: Table S1. Nurses’ attitude according to their degree of agreement toward items of FATCOD in Amhara region Referral Hospitals, Northwest Ethiopia, 2017. [file 13104_2019_4567_MOESM1_ESM.docx]

**Table S1:** Nurses’ attitude according to their degree of agreement toward items of FATCOD in Amhara region Referral Hospitals, Northwest Ethiopia, 2017.

| Statements | SD (%) | D (%) | U (%) | A (%) | SA (%) |
| --- | --- | --- | --- | --- | --- |
| Giving care to the dying person is a worthwhile experience. | 64(19.3) | 98(29.6) | 5(1.5) | 125(37.8) | 39(11.8) |
| Death is not the worst thing that can happen to a person. | 60(18.1) | 109(32.9) | 1(0.3) | 107(32.3) | 54(16.3) |
| I would be uncomfortable talking about impeding death with the dying person. | 64(19.3) | 115(34.7) | 2(0.6) | 93(28.1) | 57(17.2) |
| Nursing Caring for the patient’s family should continue throughout the period of grief and bereavement | 55(16.6) | 85(25.7) | 2(0.6) | 137(41.4) | 52(15.7) |
| I would not want to care for a dying person. | 46(13.9) | 164(49.5) |  | 77(23.3) | 44(13.3) |
| The nurse should not be the one to talk about death with the dying person. | 50(15.1) | 148(44.7) | 1(0.3) | 109(32.9) | 23(6.9) |
| The length of time required giving care to a dying person would frustrate me. | 54(16.3) | 145(43.8) | 4(1.2) | 102(30.8) | 26(7.9) |
| I would be upset when the dying person I was caring for gave up hope of getting better. | 43(13.0) | 135(40.8) | 15(4.5) | 95(28.7) | 43(13.0) |
| It is difficult to form a close relationship with the dying person. | 24(7.3) | 174(52.6) | 20(6.0) | 65(19.6) | 48(14.5) |
| There are times when the dying person welcomes death. | 62(18.7) | 84(25.4) | 27(8.2) | 134(40.5) | 24(7.3) |
| When a patient asks, “Am I dying?” I think it is best to change the subject to something cheerful | 38(11.5) | 127(38.4) | 35(10.6) | 79(23.9) | 52(15.7) |
| The family should be involved in the physical care of the dying person. | 49(14.8) | 87(26.3) | 42(12.7) | 116(35.0) | 37(11.2) |
| I would hope the person I’m caring for dies when I am not present. | 34(10.3) | 124(37.5) | 44(13.3) | 110(33.2) | 19(5.7) |
| I am afraid to become friends with a dying person. | 50(15.1) | 123(37.2) | 17(5.1) | 111(33.5) | 30(9.1) |
| I would feel like running away when the person actually died. | 50(15.1) | 135(40.8) | 3(0.9) | 116(35.0) | 27(8.2) |
| Families need emotional support to accept the behavior changes of the dying person. | 25(7.6) | 86(26.0) | 2(0.6) | 132(39.9) | 86(26.0) |
| As a patient nears death, the nonfamily caregiver should withdraw from his/her involvement with the patient. | 78(23.6) | 146(44.1) | 8(2.4) | 48(14.5) | 51(15.4) |
| Families should be concerned about helping their dying member make the best of his/her remaining life. | 50(15.1) | 32(9.7) | 17(5.1) | 168(50.8) | 64(19.3) |
| The dying person should not be allowed to make decisions about his/her physical care. | 42(12.7) | 155(46.8) | 18(5.4) | 40(12.1) | 76(23.0) |
| Families should maintain as normal an environment as possible for their dying member. | 89(26.9) | 40(12.1) | 16(4.8) | 145(43.8) | 41(12.4) |
| It is beneficial for the dying person to verbalize his/her feelings. | 75(22.7) | 66(19.9) | 21(6.3) | 131(39.6) | 38(11.5) |
| Care should extend to the family of the dying person. | 96(29.0) | 68(20.5) | 8(2.4) | 108(32.6) | 51(15.4) |
| Caregivers should permit dying persons to have flexible visiting schedules. | 52(15.7) | 87(26.3) | 3(0.9) | 160(48.3) | 29(8.8) |
| The dying person and his/her family should be the in-charge decision-makers. | 44(13.3) | 89(26.9) | 1(0.3) | 144(43.5) | 53(16.0) |
| Addiction to pain relieving medication should not be a concern when dealing with a dying person | 53(16.0) | 78(23.6) | 2(0.6) | 136(41.1) | 62(18.7) |
| I would be uncomfortable if I entered the room of a terminally ill person and found him/her crying | 77(23.3) | 66(19.9) |  | 59(17.8) | 129(39.0) |
| Dying persons should be given honest answers about their condition. | 72(21.8) | 104(31.4) | 15(4.5) | 102(30.8) | 38(11.5) |
| Educating families about death and dying is not a nonfamily caregiver responsibility. | 30(9.1) | 104(31.4) | 9(2.7) | 103(31.1) | 85(25.7) |
| Family members who stay close to a dying person often interfere with the professional’s job with the patient. | 36(10.9) | 104(31.4) | 19(5.7) | 121(36.6) | 51(15.4) |
| It is possible for nonfamily caregivers to help patients prepare for death. | 72(21.8) | 113(34.1) | 6(1.8) | 93(28.1) | 47(14.2) |
